# CRISPR to the Rescue: Advances in Gene Editing for the *FMR1* Gene

**DOI:** 10.3390/brainsci9010017

**Published:** 2019-01-21

**Authors:** Carolyn M. Yrigollen, Beverly L. Davidson

**Affiliations:** 1 The Raymond G. Perelman Center of Cellular and Molecular Therapeutics, Children’s Hospital of Philadelphia, Philadelphia, PA 19104, USA; yrigollenc@email.chop.edu; 2 Department of Pathology and Laboratory Medicine, University of Pennsylvania, Philadelphia, PA 19104, USA

**Keywords:** Fragile X syndrome 1, Fragile X-associated Tremor/Ataxia Syndrome 2, CRISPR 3, Trinucleotide Repeat 4, Gene editing

## Abstract

Gene-editing using Clustered Regularly Interspaced Short Palindromic Repeats (CRISPR) is promising as a potential therapeutic strategy for many genetic disorders. CRISPR-based therapies are already being assessed in clinical trials, and evaluation of this technology in Fragile X syndrome has been performed by a number of groups. The findings from these studies and the advancement of CRISPR-based technologies are insightful as the field continues towards treatments and cures of Fragile X-Associated Disorders (FXADs). In this review, we summarize reports using CRISPR-editing strategies to target Fragile X syndrome (FXS) molecular dysregulation, and highlight how differences in FXS and Fragile X-associated Tremor/Ataxia Syndrome (FXTAS) might alter treatment strategies for each syndrome. We discuss the various modifications and evolutions of the CRISPR toolkit that expand its therapeutic potential, and other considerations for moving these strategies from bench to bedside. The rapidly growing field of CRISPR therapeutics is providing a myriad of approaches to target a gene, pathway, or transcript for modification. As cures for FXADs have remained elusive, CRISPR opens new avenues to pursue.

## 1. Introduction

The expansion of the CGG trinucleotide repeat within the 5’ untranslated region (UTR) of the Fragile X Mental Retardation 1 (*FMR1*) gene is the predominant cause of Fragile X syndrome (FXS), and the only known cause of Fragile X-associated Tremor/Ataxia Syndrome (FXTAS) and Fragile X-associated Primary Ovarian Insufficiency (FXPOI) [1]. The trinucleotide repeat is normally between 5 and 44 CGG repeats in length and interspersed with up to 4 AGG interruptions. 

Premutation carriers have 55–200 CGG repeats which lead to misregulation of the *FMR1* gene in several ways. Carriers of the premutation allele have elevated *FMR1* mRNA levels but also have a reduction in the translational efficiency of the *FMR1* encoded protein FMRP. *FMR1* transcripts harboring premutation length CGG repeats can give rise to Repeat-Associated Non-ATG (RAN) homopolypeptides (FMRpolyA, FMRpolyG, and FMRpolyR), some of these RAN translation products with homopolymeric amino acid tracts have been shown to be toxic [2,3,4]. Additionally, RNA toxicity can result from the long CGG tracts with *FMR1* transcripts forming stable hairpin structures and binding with proteins. This aberrant protein-RNA interaction results in protein sequestration and mislocalization, impairing normal cellular processes and leading to the formation of intranuclear inclusions [5,6,7]. Premutation carriers are at risk of developing the neurodegenerative disorder FXTAS. The age of onset for FXTAS is typically greater than 55 years of age with core features including intention tremor, gait ataxia, executive dysfunction, and neuropathy. Post mortem evaluation of FXTAS patients has identified the presence of intranuclear inclusions throughout the brain [8]. Women have a lower risk of developing FXTAS but are at risk for developing the reproductive disorder FXPOI. 

Individuals with more than 200 CGG repeats, categorized as a full mutation allele, are diagnosed with FXS when this mutation becomes hypermethylated leading to a loss of FMRP. Women with FXS are often less severely affected than men; women have a protective second copy of the *FMR1* gene that is expressed in cells when the full mutation resides on the inactive X chromosome. FXS is a neurodevelopmental disorder when the full mutation allele is aberrantly methylated and transcriptionally silenced. *FMR1* epigenetic changes occur early during embryonic development and cause intellectual disability, facial dysmorphia, macroorchidism, and hyperextensible joints, which are diagnosed early in childhood [9].

Both FXS and FXTAS are neurological disorders with no disease modifying therapies. Clustered Regularly Interspaced Short Palindromic Repeats (CRISPR) is a relatively new gene editing technology which has quickly proven effective in correcting a number of pathological mutations in model systems and is advancing to clinical trials [10]. In this review, the current state of CRISPR as a tool to treat Fragile X-Associated Disorders (FXADs) will be presented.

## 2. CRISPR

The utility of CRISPR as a genome editor in eukaryotic cells was first reported in 2013 [11,12,13]; since these publications, the technology has quickly evolved and now offers a multitude of modified CRISPR associated (Cas) enzymes capable of an array of genetic modifications [14,15]. CRISPR, which was discovered as an adaptive immune system for bacteria [16], uses Cas enzymes complexed to RNA to identify invading virus and phage DNA. The “memory” mechanism, which occurred during a previous invasion with a similar species, resulted in short DNA sequences from the invaders being stored between short palindromic repeats. When these sequences are expressed into RNA, they are resolved into a structured RNA fragment that is loaded onto the Cas nuclease. The approximately 20 bp RNA sequence that corresponds to the DNA target is accessible to genomic DNA as the nuclease scans the genetic code. When the guideRNA complements with a DNA strand the nuclease changes conformation to a catalytically opened state that cleaves the foreign DNA [17]. To use CRISPR as a genome editor, the endogenous system has been adapted by using synthesized guideRNAs that direct the Cas nucleases to the genomic region of interest. After DNA cleavage, the double stranded breaks at the target site are repaired by one of the host cells’ DNA damage response mechanisms, non-homologous end joining (NHEJ) or homology directed repair (HDR) [12]. 

**Figure 1:**
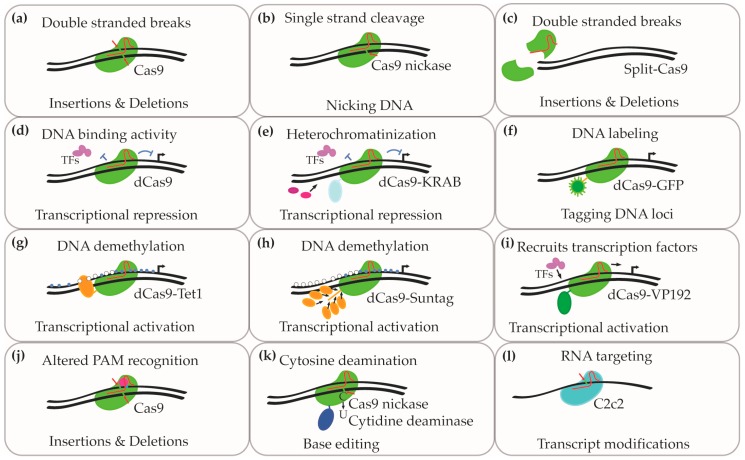
Overview of Clustered Regularly Interspersed Short Palindromic Repeats (CRISPR) technologies with therapeutic utility. (**a**) Wildtype SpCas9 is directed to the target site for editing through guideRNAs possessing sequences complementary to the DNA region of interest. When bound to the target site, the active Cas9 induces a double stranded DNA break. When repaired, this can induce the formation of indels. (**b**) Mutations that inactivate either the RuvC or HNH domain in Cas9 (Cas9 nickase) impacts nuclease such that cleavage in only one strand can occur; the other DNA strand remains intact. (**c**) Expressing split Cas9 that complexes into a functional nuclease when complementing the target DNA sequence. (**d**) Mutations in both RuvC and HNH domains deactivate Cas9; the ‘dead’ Cas9 retains the ability to bind target sequences but is incapable of generating single or double stranded breaks. When Cas9 is directed to genomic regions near start transcription start sites, expression can be inhibited because normal transcription factor binding sites are blocked. (**e**) Fusing a Krueppel-associated box (KRAB) domain onto dCas9 recruits chromatin remodeling factors that elicit heterochromatinization of the target locus, further reducing transcription (**f**) GFP fused to Cas9 is used as a molecular beacon to monitor specific regions of the genome in vitro and in vivo. (**g**–**i**) Fusing demethylases or transcriptional activators onto dCas9 to upregulate target genes. Suntag and VP192 use multiple copies of the activating domains to induce higher upregulation. (**j**) SpCas9 nuclease mutations to alter PAM recognition motifs. Wildtype Cas9 recognizes NGG, and mutation variants recognize NGCG, NGAG, NGAN, and NGNG. (**k**) Cytosine deaminase fused onto Cas9 nickase for conversion of cytosine to thymine without inducing double stranded breaks. (**l**) Cas enzymes that target RNA include C2c2, renamed Cas13a.

Cas9 from *Streptococcus pyogenes* (SpCas9) was the first CRISPR enzyme shown to edit eukaryotic DNA using synthetic guideRNAs (Figure 1a). It was successfully adapted for use in eukaryotic cells because cleavage could be achieved using only Cas9 and two short RNA molecules. Since these first reports, novel CRISPR based tools have been developed with a range of functions and advantages [14]. Modified Cas9 enzymes can now be completely deactivated from cleaving DNA while retaining their binding activity (dCas9; Figure 1d) [18,19]. When coupled to repressive proteins, the guideRNAs guide the Cas9 to the appropriate site to repress transcription (dCas9-HDAC; dCas9KRAB; Figure 1e) [20]. Variants have also been made that generate single stranded breaks (Cas9 nickases; Figure 1b) [21], increase transcription (dCas9-Suntag, dCas9-p300, dCas9-VP64, dCas9-SAM; Figure 1g–i) [20,22,23,24], label DNA (dCas9-GFP; Figure 1f), or directly convert cytosine nucleotides to thymine, or guanine to adenine (Base Editors; Figure 1k) [25,26]. Modifications to SpCas9 and other newly characterized Cas enzymes have also improved the specificity of the nuclease to a target sequence (High Fidelity Cas9) [27,28,29], altered the required protospacer adjacent motif (PAM) motifs (Figure 1j) [30,31], and demonstrated RNA targeting capability (C2c2; Figure 1l) [32].

## 3. Recently Reported CRISPR-Based Therapies

The first study published using CRISPR to edit *FMR1* targeted the full mutation allele in human induced pluripotent stem cells (iPSCs; Table 1). These cells, harboring an epigenetically silenced full mutation, were electroporated with plasmid DNA encoding SpCas9 and a guideRNA designed to target 47 base pairs upstream of the start of the trinucleotide repeat [33]. Complete deletion of the CGG repeats were observed in iPSCs. These cells, clonally expanded post-editing had a reported 2–3% editing efficiency. The edited iPSCs were shown to reactivate *FMR1* expression to levels similar to the control cells with normal CGG repeat alleles. The authors further reported a loss of methylation at the promoter following CRISPR mediated deletion of the full mutation CGG repeats, and sustained *FMR1* expression was present after reprogramming the iPSCs into neuronal cells [33]. The edited cells that were differentiated into mature neurons stained positive for FMRP compared to a lack of FMRP positive cells in the unedited parental lines. Differences in genes expressed in edited and unedited neurons showed a reduction in three glutamate receptor genes, *GRIA1*, *GRIN2B*, and *GRIN3A* following deletion of the CGG repeat, consistent with restoration of FMRP expression.

Xie and colleagues [34] used a similar strategy to delete the CGG repeats from HEK 293 cells and iPSCs using nucleofection of CRISPR plasmids with guideRNAs targeting 40 bps upstream and 35 bps downstream of the trinucleotide repeat (Table 1). With dual guides, the editing efficiency increased to 20% in iPSCs. Importantly, this study replicated that a silenced full mutation could be reactivated by deletion of the CGG repeats in iPSCs, and this transcriptional activation was stable for a prolonged time in culture (50 days post reactivation). However, it was reported by Xie et al. that not all of the clonal lines reactivated following CRISPR editing. Evaluation of epigenetic status showed that reactivated clones had decreased methylation levels at the CpG sites at the promoter region and adjacent to the CGG repeat locus, while clones that lacked reactivation showed similar methylation levels as unedited iPSC clones. This variability in epigenetic modifications and *FMR1* expression in CGG deleted cells was hypothesized to be the result of incomplete DNA remethylation following DNA replication. As such, more actively replicating cell types would be expected to undergo more efficient *FMR1* reactivation following CRISPR editing of the CGG repeat, while nondividing cell types will be less prone to this demethylation. While promising, neither study showed direct editing in differentiated cells, and further investigation into the reactivation capabilities of nondividing neurons is warranted.

CRISPR has also been used to epigenetically modify the *FMR1* full mutation outright [35]. In the first study of this kind, catalytically deactivated SpCas9 (dCas9) was fused to Tet1, an enzyme that induces demethylation of cytosines to create a methylation eraser (dCas9-Tet1). When dCas9-Tet1 and guideRNAs designed to target the CGG repeats were introduced into iPSCs with lentivirus, epigenetic changes occurred at the *FMR1* locus. As expected, gene expression occurred without sequence modifications of the trinucleotide repeat. Edited FXS iPSCs showed *FMR1* expression levels that were 90% of that measured in a control human embryonic stem cell. There was also DNA hypomethylation of the CpG island adjacent to the repeats, and histone modifications including H3 lysine 27 acetylation, H3 lysine 4 trimethylation, and a decrease of H3 lysine 9 trimethylation at the promoter of *FMR1*. The histone modifications reactivated transcription, and this was maintained for over 35 days in culture. When the edited iPSCs were derived into neurons *FMR1* remained transcriptionally active and the electrophysiological hyperactive firing rate phenotypes were rescued. Gene expression changes were described for 41 identified off target genes to be less than 4-fold upregulated in the edited neurons, while one gene *RGPD1* had a 9-fold increase in gene expression. In contrast, *FMR1* was reported to have a 481-fold increase in gene expression following epigenetic editing. When engrafted into mouse brains, the edited cells expressed FMRP for 3 months post-transplantation. This is the first in vivo analysis of ex vivo edited, transplanted neuron. These findings demonstrate the utility of targeting epigenetic-editors to the CGG repeat locus. A critical question in using gene editing technologies for FXS is whether a constitutively active dCas9-Tet1 is necessary for long term reactivation at the *FMR1* locus. Epigenetic editing of iPSC derived neurons was shown to be less efficient than in iPSCs, *FMR1* mRNA levels were restored to 45% that of control neurons and a 20% decrease in CpG methylation at the *FMR1* promoter was achieved post editing. These differences could have been the technical limitations of isolating the edited neurons or differences in mechanisms to demethylation between cell types. The authors also reported a rescue of hyperactivity in the edited FXS neurons compared to their unedited counterparts.

In another approach, Haenfler et al. [36] fused dCas9 to multiple VP16 transcriptional activator domains (dCas9-VP192) to drive expression of *FMR1* without altering the genetic sequence (Table 1). Human embryonic stem cells harboring a silenced full mutation allele were transfected in vitro with dCas9-VP192 and guideRNAs targeting either the *FMR1* promoter region or the ~800 CGG trinucleotide repeats. Cells transfected with the transcriptional activator targeting the CGG repeats showed robust transcriptional reactivation but only modest gains in FMRP. The CGG targeting guideRNAs were reported to induce higher transcriptional activity than guideRNAs that targeted the promoter region. The ability of the CGG targeting guideRNAs to target within the trinucleotide repeats multiple times might explain the stronger reactivation of *FMR1*, as more VP192 activating domains are recruited to the gene. They also transfected neuronal progenitor cells derived from the fully methylated hESC with the CGG targeting dCas9-VP192 and reactivated *FMR1* and increased transcriptional expression. Again, there was a limited increase in FMRP. This work highlights a key limitation of reactivating a full mutation; long CGG repeats have been shown to decrease the translational efficiency of *FMR1* [37]. The molecular implications of expressing long CGG repeat tracts is also an area that needs further investigation, as 55–200 repeats can give rise to FXTAS and individuals with an unmethylated full mutation have been reported to develop FXTAS symptoms [38,39]. Thus it will be important to understand if expressing more than 200 repeats will increase the risk of developing FXTAS or increase the severity of clinical pathology.

The first in vivo gene-editing in animal models of FXS was recently reported by Lee et al. This strategy assessed both Cas9 and Cpf1 for their efficiency to knockout gene expression in vivo in mice. The nucleases were delivered to specific regions of the brain using gold-nanoparticles with editing in neurons, astrocytes, and microglia shown to decrease reporter gene expression. In an alternative gene editing strategy to targeting *Fmr1*, the metabotropic glutamate receptor (mGluR5) encoding gene *Grm5* was targeted for knockdown with Cas9 in the striatum of *Fmr1* knockout mice (Table 1) [40]. *Grm5* was targeted because of the over-activation of mGluR5-dependent signaling present in FXS and other autism spectrum disorders. The efficiency of editing *Grm5* was measured to be 14.6%, resulting in a 40–50% reduction in the encoded protein. Repression in mGluR5-dependent signaling resulted in a rescue of a marble burying phenotype in *Fmr1^-/y^* mice as well as jumping behaviors, both considered measures of hyperactivity and stereotypy. While this study demonstrated in vivo gene-editing in an animal model of FXS, it did not target the *FMR1* locus directly. However, the work did establish a potential strategy for treating autism. Although the authors used CRISPR editing to treat FXS-like phenotypes in mice, they did so by inducing a loss of function mutation in a second gene rather than correct the initial null mutation in *Fmr1*; the authors suggest that while clinical trials that used drugs to reduce mGluR5 activity were disappointing, using CRISPR to target the overactivation may be more effective. Of note, the delivery strategy resulted in a focused treatment area, which is necessary when knocking out an important regulator of plasticity. However, it has limitations if multiple regions or a large area of the brain must be edited for therapeutic effect, an important consideration in translating from mice to humans. Notably, gold nanoparticles have been shown to have cellular toxicity at high or repeated doses [41,42].

Other trinucleotide repeat disorders are also being targeted with CRISPR-based therapies [43]. One example is the Huntingtin gene (*HTT*), which has been corrected in vitro using patient derived fibroblast cells by targeting only the expanded allele for deletion of the CAG repeat and knocked down expression. This strategy was also used in vivo in a mouse model of the expanded CAG repeat, with similar decreased expression of the gene and encoded protein as was seen in cell lines [44]. An in vivo mouse model of Friedreich Ataxia showed partial increased expression of the frataxin (*FXN*) gene when GAA trinucleotide repeats were deleted from intron 1 with Cas9 [45].

The modifications, fusions, and evolutions of CRISPR for therapeutics opens the field to numerous strategies for using gene-editing to treat and cure FXADs. For example, RNA targeting nucleases can be used to target the CGG repeat in FXTAS and off-target activity can be decreased using split Cas9 or Cas9 nickases that require two guideRNAs to complement the genomic sequence near each other [46,47]. The SpCas9 nuclease has also been genetically modified to decrease off-target activity while maintaining high editing activity at the target loci [28,29]. These designer nucleases can improve the safety of these therapies by minimizing unwanted DNA damage. Cas9 enzymes with alternative PAM motifs are also available, and these make more of the genome accessible for CRISPR modification [31].

## 4. Further Considerations for Gene-Editing for FXTAS and FXS

### 4.1. Delivery

Delivering CRISPR-based therapeutics into the brain and specifically neuronal cells is essential for FXS and FXTAS therapy. Several strategies to deliver gene therapies to the brain, some with success in model systems, are described below. Also see a more comprehensive review by Cwetsch, Pinto [48].

Transducing neurons with Adeno Associated Virus (AAV) is the most common method to deliver CRISPR editors in vivo. AAVs have high transduction efficiency and low immunogenicity profiles compared to many other viral vectors, contributing to their broad use in gene therapy applications [49]. AAVs can package approximately 4.8 kb of foreign DNA sequence, which is only 600 bp larger than the coding sequence of SpCas9. This leaves minimal room for cassettes to drive guideRNAs and Cas9 expression. Strategies to bypass this limitation include dual vector systems where the nuclease and guideRNAs are packaged into separate AAV vectors [50] or splitting Cas9 into two fragments that dimerize in the presence of a small molecule (Figure 1c) [51]. Alternative enzymes that are encoded by sequences smaller than the 4.2 kB sequence for SpCas9 include the 3.2 kB *Staphylococcus aureus* Cas9, which is small enough to allow for cis expression of one or two guideRNAs [30,52].

AAV capsids vary in their tropism, defined as their ability to bind to and be internalized by specific cell types. AAV serotype 9 has been used to deliver an *FMR1* transgene into *Fmr1* knockout neonate mice via intracerebroventricular injection [53]. These experiments showed neuronal expression of FMRP in several brain regions including the cortex, hippocampus, and striatum. The cortex and hippocampus had the highest transduction efficiency as measured by the number of FMRP-positive cells for a given area. However, FMRP was not detected beyond the midbrain and cerebellum. Together with AAV9, AAV1, AAV2, AAV5 and AAV8 have been reported to express transgenes throughout the brain [54,55]. Developing genetically engineered AAV serotype variants that increase overall transduction efficiency or specificity is an active area of research [56,57].

Lentiviruses have also been engineered to deliver CRISPR components into cells. Unlike AAVs, that remain mostly episomal; lentiviruses integrate into the host genome, a mechanism that can increase the risk of insertional mutagenesis. An advantage of lentiviruses for gene editing is that they have a packaging capacity of approximately 9 kb. Lentiviruses have been used to deliver CGG targeting CRISPR components into iPSCs in vitro for gene editing as discussed above [35].

Lipid nanoparticles (LNPs) are a non-viral strategy to deliver CRISPR components in vivo. In the liver, high editing efficiency was shown after a single intravenous injection of CRISPR-mRNA containing LNPs [42], or LNPs containing plasmids encoding Cas9 and guideRNAs [58,59]. Currently, efficient editing in the brain using LNP-based systems have not been shown. However, *Fmr1* knockout mice have been focally transfected with gold nanoparticles, as described above [40].

The main advantage of non-viral delivery is that the Cas nuclease is present and functional for a limited time. In recent studies comparing plasmid expression systems or RNPs of Cas9/guide RNAs, Behlke and colleagues showed that transient Cas9 exposure minimizes off target activity [60]. Additionally, transiently expressing Cas and guideRNAs provides the availability of the full CRISPR toolkit for editing new targets without interference from a previous treatment.

The route of CRISPR delivery into the brain has to be considered. Some modalities will allow for widespread delivery but require larger dosages to be administered (i.e., intraventricular injection) while others can provide for targeting a precise region (i.e., intracerebellar or cerebral injection). Localized injections will minimize the amount of therapeutic administered while having a high coverage of cells in the targeted area. Such a delivery strategy will also narrow the functional range of the therapy. This is unlikely to be useful for FXS and FXTAS therapy where pathology occurs in many brain regions [61,62]. FXS is associated with increased white matter and gray matter volume in the thalamus, frontal and temporal lobe, cerebellum and caudate by magnetic resonance imaging (MRI). Histopathological analysis of brains from individuals with FXS identified abnormalities in the hippocampus and cerebellar vermis [63]. Patients with FXTAS often have increased T2 intensity by MRI in the middle cerebellar peduncles (MCPs), this is referred to as the MCP sign and occurs in approximately 60% of diagnosed cases of FXTAS. Other hallmarks of the disorder include cerebellar and cerebral atrophy and a thinning of the corpus callosum. Hippocampal and amygdala structural differences are less obvious and not consistently seen across studies. Balancing precision and spread of the therapeutic components will be important for successful clinical results, and will be heavily dependent on the delivery vehicle [64].

Another important consideration is when to deliver the gene-editors. The timeframe for treatment will be different depending on whether FXS or FXTAS is being treated. While FXS symptoms occur early during development and early interventions show the greatest outcomes [65], FXTAS is a late onset and incompletely penetrant disorder. Premutation carriers may never develop FXTAS or require medical intervention, but we are yet unclear whether features of FXTAS can be improved post onset and what treatment windows will be beneficial. 

### 4.2. Off-Target Editing

The main concerns of researchers developing CRISPR therapies are the off-target and undesired editing effects permanently incorporated into patients’ somatic cells. Off target editing has been studied in vitro and in vivo using a variety of genome-wide protocols (i.e., Genome-wide, Unbiased Identification of DSBs Enabled by Sequencing [GUIDE-seq] [66]; High-Throughput Genome-Wide Translocation Sequencing [HTGTS] [67]; Integrative-Deficient Lentiviral Vectors [IDLV] [68]; Digenome-sequencing [Digenome-seq] [69,70]; Circularization for In vitro Reporting of Cleavage Effects by sequencing [CIRCLE-seq] [51]; Selective enrichment and Identification of Tagged genomic DNA Ends by sequencing [SITE-seq] [71]; Breaks Labeling, Enrichment on Streptavidin, and Sequencing/Breaks Labeling In Situ and Sequencing [BLESS/BLISS] [30,72,73], and others [74]). These off-target detection screens have demonstrated that prediction of sites prone to erroneous cleavage by CRISPR is challenging and impacted by the guideRNA sequence, tissue or cell type, the specific nuclease used, and the method of delivery. Off-target activity is balanced with on-target editing efficiency. A high on-target, low off-target ratio is of course the holy grail of therapeutic editing. To reduce off-target binding of guide RNAs, most current guideRNA design tools use algorithms that allow minimizing the number of mismatches of off-target sequences in the host genome to the on-target sequence. Some algorithms also consider chromatin accessibility and guideRNA stability in vivo, although this information changes with cell type [75,76,77]. 

Characterizing and reducing off-target effects are critical to move any gene-editing therapy into the clinic. None of the recently published *FMR1* editing studies have surveyed off-target editing using one of the genome wide unbiased methods [51,66,68,69,72,73], with predicted off-targets shown to have little overlap with off targets identified using these approaches. Off-target loci have been shown to occur with as many as 6 mismatches to the 20 nucleotide guideRNA sequence [78], and are significantly reduced with transient Cas9 expression, either by delivery of mRNA encoding the nuclease and guideRNAs, or delivery of the complexed RNPs [79]. Self-destroying CRISPR (KamiCas9) systems has also been shown to limit the time Cas9 is present in treated cells, reducing off-target accumulation [80].

Editing systems must be empirically tested for each gene, and possibly for each cell type, of interest. In general, multiple guideRNAs are tested and their on- and off-target editing efficiencies evaluated. A consideration of off-target effects is whether a biological consequence (e.g., truncated protein) would result from misdirected editing and how well such an outcome is tolerated by the cell type.

CRISPR-editing of the *FMR1* gene may erroneously repair the on-target sequence resulting in large deletions, incorporation of plasmid or viral DNA sequences, or fusion of *FMR1* with other genes at other loci. A non-biased genetic analysis of repaired sequences will quantify which mistakes are prone to occur and at what frequency. Large deletions extending beyond the start codon, or upstream of the transcriptional start site, would disrupt *FMR1* expression or function. Incorporation of foreign DNA sequences may dysregulate *FMR1* reducing therapeutic rescue post editing [81,82]. Fusions of genes after editing could create novel, immunogenic or toxic proteins or protein chimeras that could alter the function of the cell or even be tumorigenic [83,84]. While these possibilities seem dire, careful preclinical testing in relevant model systems would identify problematic editing components such that alternative approaches could be developed and tested.

One of the unique considerations of gene-editing for FXADs therapy is the presence of functional and dysfunctional CGG repeats in females. Females are genetically protected by the presence of a normal CGG repeat allele in a portion of their cells [85,86]. It is important to avoid editing the normal allele-containing cells, while targeting premutation or full mutation expressing cells. Liu et al suggest that preferential editing at the expanded allele occurs because the CGG repeats provide more targetable sequence compared to the few CGG repeat in the normal allele or at other genomic loci Liu, Wu [35]. While possible, this requires more thorough investigation using single cell RNA seq analysis of cells after CRISPR/Cas9 editing of a mixture of cells harboring normal and expanded repeat sequences.

### 4.3. CRISPR Limitations

There are several additional limitations when implementing the CRISPR technology as a therapeutic strategy. The stringency of CRISPR nucleases to a PAM sequence can prevent researchers from targeting a specific genomic locus because there are no available PAM sites within that region, this results in changing the gene-editing strategy to conform to the currently accessible sequences. However, CRISPR tools are being developed to broaden the sites that Cas9 and other nucleases recognize [31]. The incomplete editing of CRISPR in somatic cells can result in mosaicism of desired and off target modifications in vivo. These mosaic modifications can result in unpredictable outcomes [87]. As the field continues to progress, we will have an improved perspective on how often these sporadic events occur and the severity they have in vivo. 

### 4.4. Ethical Considerations

With the rapid progress of CRISPR in the biomedical field, keeping pace with the ethical issues that arise is important. Like clinical trials that are currently underway for new pharmaceuticals and gene therapies, gene-editing has the potential for unforeseen outcomes and side effects. Because the therapy is a permanent modification of the genome, these unexpected effects can carry a greater burden to the participant. Treatment of an embryo, child, or patient incapable of understanding the risks associated with the therapy mean informed consent cannot be obtained. In a disorder as variable as FXS, balancing the potential benefit of treating a patient during early development with the ability to assess the severity of the individual’s symptoms later in life comes into question. Mosaic off-target mutations can result in unpredictable side effects to the therapy in a single participant. Additionally, as access to gene-editing therapies becomes available, equal access to these technologies across society will be essential to not disenfranchise sectors of our society [88]. The ethical issues that arise as genome editing in humans continues to advance are important and more each come with serious consequences can result from how these issues are resolved. As we move forward with developing genome editing therapies that have the potential to greatly improve the quality of life of individuals within the Fragile X community, it is important to also move forward in our understanding of these issues and decide how to address these ethical considerations.

## 5. Final Remarks

The recent successes that have been achieved using CRISPR for genetic and epigenetic editing and the advances being made to deliver, control, and modify Cas nucleases are driving rapid development of editing-based gene therapies for disease treatment. The continued developments in the field, even for other disorders, will allow us to optimize CRISPR technology for FXADs with regards to efficacy and safety. Finally, CRISPR/Cas9 technologies may also provide tools to develop an in vivo model of the *FMR1* epigenetically silenced full mutation, which to date does not exist. Thus, the technological advancement of CRISPR/Cas9 has infiltrated many sectors of biomedical research and provides one of the more promising approaches for FXS and FXTAS therapy.

## Figures and Tables

**Table 1:** Summary of Fragile X syndrome (FXS) CRISPR gene editing studies.

Study	Cells or Tissue	Host	Delivery	Target Sequence	Nuclease Used	Outcome
Park et al., *Cell Reports* 2015	iPSC	Human	Electroporation	47 bp upstream of CGG repeat	SpCas9	Deletion of CGG repeats in 2–3% clonally expanded cells; reactivation of the *FMR1* gene; sustained *FMR1* reactivation upon differentiation of iPSCs into neurons.
Xie et al., *PLoS ONE* 2016	iPSC	Human	Nucleofection	40 bp upstream and 35 bp downstream of CGG repeat	SpCas9	Deletion of CGG repeats in 20% of cells; variability in reactivaton among edited clones. Reactivation of *FMR1* persisted 50 days in culture.
Liu et al., *Cell* 2018	iPSC	Human	Lenti virus	CGG repeats	dCas9-Tet1	Reactivation of *FMR1* and demethylation of the CGG repeat locus in iPSCs and derived neurons. Lower reactivation efficiency in neurons. Edited iPSCs were reprogrammed into neurons and engrafted into mouse brain; half of neurons actively expressed FMRP 3 months post transplantation.
Haenfler et al., *Frontiers of Molecular Neuroscience* 2018	ESC and neuronal progenitor cells	Human	Lipid mediated transfection	Promoter and CGG repeats	dCas9-VP192	Reactivation of *FMR1* but low FMRP expression with CGG targeting. Higher reactivation efficiency with CGG targeting guideRNAs than promoter targeting.
Lee et al., *Nature Biomedical Engineering* 2018	Striatum	Mouse	Gold nanoparticles	*Grm5*	Cas9 and Cpf1	Reduction of *Grm5* expression; phenotypic rescue of marble burying and jumping behaviors.
